# Artificial Intelligence for Prognostic Modelling and Adaptive Treatment Monitoring in Radiation Oncology

**DOI:** 10.7759/cureus.110105

**Published:** 2026-06-02

**Authors:** Krishna Chidrawar, Sandeep Kaur Toor, Shubham Gupta, Varsha Mary Khalkho, Abhishek Anand, Pallavi YC

**Affiliations:** 1 Department of Radiology, Durga Diagnostic Centre, Maharashtra University of Health Sciences, Nashik, IND; 2 Department of Radiodiagnosis, Punjab Institute of Liver and Biliary Sciences, Mohali, IND; 3 Department of Radiodiagnosis, Government Medical College Jammu, Jammu, IND; 4 Department of Radiology, School of Medical Sciences, Sri Satya Sai University of Technology and Medical Sciences, Sehore, IND; 5 Department of Pharmacy Practice, Teerthanker Mahaveer College of Pharmacy, Teerthanker Mahaveer University, Moradabad, IND; 6 Department of Agadatantra and Vidhi Vaidyaka, Rajiv Gandhi University of Health Sciences, Bengaluru, IND

**Keywords:** adaptive radiotherapy, artificial intelligence, deep learning, prognostic modelling, radiomics

## Abstract

Artificial intelligence (AI) is increasingly being used in radiation oncology to help doctors predict patient outcomes and monitor treatment response. However, its routine use in clinical practice is still limited because available studies are not always consistent, methods are not fully standardised, and many AI tools have not been tested widely in real-world settings. This narrative review examines how AI is used for prognosis, adaptive radiotherapy, treatment response assessment, and toxicity prediction in radiation oncology. This review also considers AI applications in cancer screening, radiological diagnosis, and radiology-histopathology correlation, as these areas directly support prognostic modelling and adaptive treatment decisions. A structured literature search from 2015 to 2025 was performed across major biomedical databases, with attention to radiomics, machine learning, deep learning, response modelling, and adaptive treatment planning. Studies were reviewed for their design, validation methods, and clinical outcomes. Current evidence suggests that AI can improve risk prediction, support automatic tumour and organ segmentation, track changes during treatment, and identify early signs of toxicity better than some conventional approaches. However, many studies still lack external validation and multicentre data. Challenges also remain in making AI models easy to understand and compatible with existing clinical systems. Combining imaging data with genomic information and radiation dose parameters may further improve prediction. In clinical practice, AI may help personalise radiation dose, support timely treatment plan adjustment, and improve resource use. Wider adoption will require stronger validation, standardised workflows, and clear model governance. Overall, AI should be used as a decision-support tool to assist clinicians rather than replace clinical expertise.

## Introduction and background

Radiation oncology increasingly depends on accurate imaging, pathology correlation, and longitudinal treatment assessment to guide patient-specific radiotherapy decisions. In this setting, artificial intelligence (AI) has moved beyond cancer detection alone and is now being studied for prognosis, target delineation, adaptive treatment planning, response monitoring, and toxicity prediction. The scope of this review is therefore focused on AI applications that support radiation oncology workflows, particularly prognostic modelling and adaptive radiotherapy. Screening and radiological diagnosis are discussed only where they contribute to treatment selection, baseline risk stratification, or radiology-histopathology correlation before radiotherapy. Adaptive radiotherapy refers to modifying a radiation treatment plan during the treatment course when tumour size, patient anatomy, or organ-at-risk position changes.

Cancer remains a major cause of morbidity and mortality in the United States, creating a need for more precise methods to detect disease, define tumour extent, estimate prognosis, and monitor treatment response during oncology care [1]. AI-assisted screening studies have shown that computational tools can help identify cancers that may be missed during routine image interpretation [2]. At the same time, machine learning and deep learning methods are increasingly being applied to imaging data to improve cancer detection, staging, and outcome prediction [3]. Breast cancer imaging has provided important early evidence for AI-based lesion detection and classification, although screening alone is not the central focus of this review [4]. More broadly, AI-powered medical imaging can support national cancer care systems by improving diagnostic consistency, risk stratification, and treatment planning [5].

Although cancer screening studies provide useful evidence for AI-assisted image interpretation, they are included in this review only when they inform radiation oncology workflows. For example, automated lesion detection, image-based triage, and workload reduction may improve baseline assessment before radiotherapy [6]. Public imaging repositories have also supported the development of AI models, but demographic underrepresentation in these datasets may limit fairness and generalisability [7]. Deep learning models trained on medical imaging data can extract complex features that may support prognosis and treatment response assessment beyond visual interpretation alone [8]. Digital pathology adds another important layer by allowing AI-based analysis of tissue morphology and radiology-histopathology correlation [9].

Traditional radiological and pathological assessment can be time-consuming and may vary between observers, especially when tumour margins, treatment response, or subtle tissue changes are difficult to define. AI methods can help address these limitations by converting clinical images and pathology slides into structured quantitative data. Radiomics extracts measurable features from standard images, including tumour shape, intensity, texture, and heterogeneity. Machine learning can then use these features to identify patterns linked with survival, recurrence, treatment response, or toxicity. In radiation oncology, these tools may support target segmentation, organ-at-risk assessment, dose adaptation, toxicity prediction, and follow-up monitoring.

Radiology-histopathology correlation is relevant because imaging-based AI models require biological and clinical validation. Linking radiomic features with histopathological findings, molecular markers, dosimetric data, and patient outcomes can help determine whether image-derived patterns reflect tumour aggressiveness, treatment sensitivity, or normal tissue vulnerability. This integration is important for developing clinically meaningful prognostic biomarkers rather than isolated algorithmic outputs.

Despite these opportunities, several barriers limit routine clinical use. Many AI models are trained on retrospective datasets, have limited external validation, and may not generalise across scanners, institutions, or patient populations. Existing clinical workflows may also lack the capacity to combine primary cancer findings, non-cancer comorbidities, imaging changes, and longitudinal treatment data in a single decision-support pathway [10]. Therefore, AI systems for adaptive treatment monitoring must be evaluated within real radiation oncology workflows rather than only as stand-alone diagnostic tools.

This review evaluates the role of AI in prognostic modelling and adaptive treatment monitoring in radiation oncology. It specifically examines imaging-derived biomarkers, machine learning and deep learning survival models, multimodal integration, baseline risk stratification, early response prediction, longitudinal tumour monitoring, adaptive radiotherapy planning, and toxicity prediction. Screening, radiological diagnosis, and histopathological correlation are considered only as supporting domains when they inform prognosis, treatment planning, or adaptive radiotherapy decisions.

Objectives of the review

This review aims to answer the following question: In patients with cancer undergoing evaluation or treatment within radiation oncology workflows, how are AI approaches, including radiomics, machine learning, and deep learning, applied to imaging modalities such as computed tomography (CT), magnetic resonance imaging (MRI), positron emission tomography (PET), ultrasound, mammography, and histopathology-linked imaging data to support prognostic modelling, adaptive radiotherapy monitoring, treatment response assessment, and toxicity prediction?

The review focuses on radiation oncology and radiotherapy-related clinical decision-making, while radiological diagnosis, screening, and histopathological correlation are considered only when they inform baseline risk stratification, treatment planning, or adaptive treatment monitoring. It evaluates the integration of AI-based tools into adaptive radiotherapy workflows, including tumour and organ-at-risk segmentation, longitudinal response assessment, toxicity forecasting, and dose adjustment. The review also examines methodological trends, validation practices, and translational barriers to clinical implementation. Finally, it identifies gaps requiring standardisation, external validation, multicentre prospective evaluation, and clearer integration into routine radiation oncology practice.

For clarity, classification refers to assigning cases to predefined groups, detection refers to identifying disease presence or location, and time-to-event modelling estimates outcomes such as survival or recurrence over follow-up. The C-index is commonly used for survival models, while the area under the curve (AUC) is commonly used for classification or detection tasks.

Search strategy and study selection

A structured literature search was conducted to identify studies published between January 2015 and December 2025 that examined the use of AI in radiation oncology, radiotherapy planning, prognostic modelling, treatment response assessment, and adaptive treatment monitoring. The main databases searched were PubMed/MEDLINE, Scopus, Web of Science, and Google Scholar. Additional relevant articles were identified by reviewing the reference lists of selected papers.

The search used combinations of the following keywords and Boolean operators: "artificial intelligence" OR "machine learning" OR "deep learning" OR "radiomics" AND "radiation oncology" OR "radiotherapy" OR "adaptive radiotherapy" AND "prognostic modelling" OR "survival prediction" OR "treatment response" OR "toxicity prediction" OR "treatment monitoring". Additional terms such as "CT", "MRI", "PET", "segmentation", "dose planning", "organ-at-risk", "histopathology", and "radiology-pathology correlation" were used where relevant.

Articles were considered eligible if they focused on AI-based methods applied to cancer imaging, radiotherapy workflows, adaptive treatment planning, response monitoring, survival prediction, or toxicity assessment. Studies were prioritised if they reported clinical outcomes, model performance metrics, validation strategies, or implementation barriers. Exclusion criteria included non-oncology studies, purely technical studies without clinical relevance, opinion articles without supporting evidence, studies not available in English, and articles unrelated to radiation oncology or radiotherapy-related decision-making.

As this manuscript is a narrative review, formal systematic review tools for study quality assessment or risk-of-bias scoring were not applied. The search was completed in December 2025, and only English-language articles were considered. Titles and abstracts were screened for relevance to radiation oncology, radiotherapy workflows, prognostic modelling, adaptive treatment monitoring, treatment response, or toxicity prediction. Full texts were then reviewed to extract information on study design, cancer type, imaging modality, AI method, clinical endpoint, validation approach, performance metric, and implementation relevance. Evidence was synthesised narratively by grouping studies according to clinical function, including prognostic modelling, baseline stratification, response monitoring, adaptive radiotherapy planning, toxicity prediction, and clinical implementation. Because this was designed as a narrative review rather than a systematic review, a formal Preferred Reporting Items for Systematic Reviews and Meta-Analyses (PRISMA) flow diagram was not prepared. Formal meta-analysis or pooled statistical synthesis was not performed because of substantial heterogeneity across imaging modalities, AI architectures, datasets, validation methods, clinical endpoints, and reporting approaches among the included studies.

## Review

Imaging-derived prognostic biomarkers: from morphology to quantification

Quantitative imaging biomarkers allow clinicians to stratify patients according to tumour aggressiveness, which allows more granular prognostic evaluation than qualitative reporting [1]. Deep learning models can analyse huge datasets and extract sub-visual hierarchical features, which can help to recognise complex features within cancerous lesions [5]. Manual histopathological assessment, defined as the microscopic examination of tissue samples by a pathologist, and radiological assessment can both be prone to inter-observer variation and false-negative findings during screening [6]. The computerisation of the tissue slides into density matrices permits the analysis of the computer cell types, resolving issues with manual microscopic inspection [9].

Automated deep learning radiomic biomarkers derived from baseline CT scans have shown moderate prognostic performance for overall survival, with reported C-index values of approximately 0.6-0.7 in real-world and clinical trial validation settings [11]. This suggests that such biomarkers may add prognostic information beyond conventional size-based measurements, although their clinical value requires further validation. Principal component analysis, an unsupervised dimensionality reduction method, has been used to reduce high-dimensional radiomic features before survival modelling. This approach may improve model stability by limiting redundancy and preserving major patterns of variation, but it should be interpreted cautiously because feature reduction can also remove clinically relevant information [12]. Multi-feature radiomic signatures have been identified as independent prognostic factors for early-stage malignancies with an incremental predictive value compared to conventional TNM staging systems [13-15]. Standardised quantitative evaluation of tumour phenotypes to provide initial insight into survival from the pre-treatment imaging data is called baseline radiomic signatures [14].

The step from descriptive morphology into quantitative analytics enables the information yield of high-dimensional feature sets that are more closely reflected in their underlying tumour biology and prognosis [16,17]. While manual quantification is limited by human interpretation, automated computational pipelines are a robust and reproducible way of identifying sub-visual biomarkers for outcome prediction [6,9,12,16]. Table 1 summarises AI-based prognostic models using imaging, histopathology-linked, multimodal, and clinical registry datasets. The task categories have been revised to distinguish image-based classification, detection, and time-to-event prognostic modelling. Clinical registry datasets are listed separately from imaging modalities to avoid implying that Surveillance, Epidemiology, and End Results (SEER) data are imaging-based.

**Table 1:** AI-based prognostic modelling, survival prediction, and risk stratification by data source AI: artificial intelligence; CT: computed tomography; MRI: magnetic resonance imaging; T1CE: T1-weighted contrast-enhanced; T2 FLAIR: T2-weighted fluid-attenuated inversion recovery; DWI: diffusion-weighted imaging; PET: positron emission tomography; RWD: real-world data; CNN: convolutional neural network; LASSO: least absolute shrinkage and selection operator; Cox: Cox proportional hazards model; AUC: area under the curve; DFS: disease-free survival; OS: overall survival; NSCLC: non-small cell lung cancer; PD-(L)1: programmed death (ligand) 1; IHC: immunohistochemistry; SBMU: Sri Balaji Medical University; SEER: Surveillance, Epidemiology, and End Results; Nnet: neural network Table 1 is organised by data source and modelling purpose. Classification assigns cases to predefined groups, detection identifies disease on imaging, and time-to-event modelling estimates outcomes such as survival over follow-up. C-index evaluates survival-model discrimination, while AUC evaluates classification or detection performance; these metrics should not be interpreted interchangeably.

Data source/modality	Dataset used	Model type	Task category	Performance metrics	Key findings/predictors identified	Reference
CT/PET	RWD + clinical trial data	3D CNN + Cox model	Time-to-event prognostic modelling/treatment response prediction	C-index: 0.6-0.7	Predicted survival and PD-(L)1 response using imaging-derived biomarkers	[11]
Contrast-enhanced CT	Retrospective cohort (n=282)	LASSO Cox radiomics	Time-to-event prognostic modelling	C-index: 0.72	Radiomics signature predicted disease-free survival	[13]
Contrast-enhanced CT	Saint-Luc cohort	Cox delta-radiomics	Time-to-event prognostic modelling	C-index: 0.68	Delta-radiomics supported overall survival prediction in NSCLC	[14]
Clinical registry data	SEER population registry	DeepSurv	Time-to-event prognostic modelling	C-index: 0.71	N stage and tumour grade were key survival predictors	[15]
Clinical registry data	SEER population registry	Random survival forest	Time-to-event prognostic modelling	C-index: 0.72	Hormonal status and nodal features contributed to survival prediction	[15]
Histopathology/IHC-linked clinical data	SBMU registry	Nnet-survival	Time-to-event prognostic modelling	C-index: 0.77	Stage, age, and lymph node status predicted survival	[16]
MRI + multi-omics	Discovery cohort	Multi-omics model	Survival prognostication	AUC: 0.78	Combined clinical, radiomic, and genomic data improved prognostic modelling	[18]
MRI (T1CE, T2 FLAIR, DWI)	105 patients	Fusion radiomics	Molecular/imaging-based classification	AUC: 0.925	Tumour and peritumoral oedema signatures supported molecular classification	[19]
MRI (glioma)	Institutional cohort	Random forest	Imaging-based classification/prognostic stratification	AUC: 0.92	Age, histogram, and texture features supported risk stratification	[19]

Machine learning frameworks for time-to-event prediction

The development of machine learning in oncology has moved from basic regression-based tools toward deep learning architectures that can identify sub-visual prognostic patterns in medical images [8]. Standard radiomic pipelines combine deep learning feature extractors and Cox proportional hazards models for generating a continuous risk score for estimating progression-free survival on advanced malignancies [11]. High-dimensional radiomic datasets often contain a very large number of imaging features compared to the number of patients available for analysis, a limitation commonly called the "large P, small N" problem. This imbalance can make prediction models unstable or prone to overfitting, requiring feature reduction and subsampling methods to improve the reliability of survival-based models [12]. Computational nomograms using multi-feature signatures offer more granular estimations of survival and more calibration than assessment systems based on a single risk factor [13].

Survival-specific machine learning methodologies, such as random survival forests, are a straightforward extension of ensemble learning to the time-to-event data distribution, in which censoring and time-dependent covariates are directly incorporated into the process of training such models [15]. Discrete-time survival models for neural networks use discrete sets of time intervals to estimate the joint distribution of survival time and events in the right-censored nature of clinical data [16]. Neural survival networks trained in continuous dataset frameworks, such as CoxTime, make use of specific types of loss lifecycle models to model complex, not so linear treatment interactions without sharing the proportionality assumptions of traditional statistical modelling [17]. Simple multilayer perceptron-based deep neural network models are shown to have better predictive accuracy and discriminatory power in estimating long-term survival than conventional Cox regression models [18].

While the traditional Cox regression is a baseline method for estimating the hazard, neural survival frameworks and ensemble methods such as interaction forests are a way to develop more capacity to capture non-linear interactions and complex data structures inherent in oncologic datasets [17,18]. Modern survival analysis in radiology has focused on computational architectures based on factors specific to survival analysis, such as employing loss functions or discrete-time modelling designed for survival analysis to handle censored data, thus achieving better ranking of events and enhancing stratification of risk [17].

Multimodal prognostic integration across radiology and oncology

The development of multimodal combinations of the imaging, genomic, and histopathological data offers a superior characterisation of tumour biology compared to assessment by single modalities [1]. The integration of imaging features extracted from AI into national cancer surveillance systems must have robust socio-technical integration into analytics-capable enterprise environments to realise the diagnostic value at the population level [5]. The combination of spatial cellular relationships with transcriptomic and genomic datasets in digital pathology has made it possible to identify imaging surrogates for the tumour microenvironment [9]. The addition of clinical parameters, including the age of the patient and manual measurements of the lesion, into the radiomic pipeline of deep learning models can yield incremental predictive gain in an estimate of survival [14].

Multi-habitat MRI radiomic models incorporating the tumour core together with peritumoral oedema, which refers to swelling and fluid accumulation in tissues surrounding the tumour, provide improved accuracy in determining molecular signatures when combined with genetic sequencing data [19]. The application of multimodal approaches for oncologic care depends on the synergy between the use of biological specific markers and adaptive treatment planning for better patient-specific outcomes [20]. Integration of molecular profiling and metabolic imaging results of amino acid radiotracers offers an added prognostic layer in the definition of the boundary of infiltration for high-grade malignancy [21]. Multimodal neuroimaging models are able to accurately classify overall survival, including contralesional structural cortical thickness and functional connectivity correlation, using preoperative resting state networks [22].

Cross-domain prognostic integration, including the fusion of high-dimensional sets of imaging features with molecular, genomic, and perioperative clinical variables, is repeatedly shown to show incremental predictive improvement over models based on single-measure models [1,21]. Successful multimodal integration in oncology requires the coordination of the hierarchical computational features and the structured multi-omics data to offer a robust and holistic assessment of clinical risk [5,22].

Baseline risk stratification at treatment initiation

AI-based triaging systems are able to classify screening mammography into workstreams that will aid in the enhanced assessment of patients with the highest risk of harbouring sub-visual interval cancers [2]. Radiomic signatures permit improved pre-treatment risk stratification in early-stage lung cancer, to identify patients who may request more intensive therapeutic interventions, according to the predicted risk of recurrence [13]. Preoperative radiomic analysis of baseline structural MRI may help predict molecular genotype non-invasively and support surgical planning, including decisions about tumour margins and extent of resection [19]. Comprehensive baseline risk stratification should also consider tumour location, molecular profiles, and tumour-host interactions to reduce clinical variability and support more consistent treatment planning [20].

Baseline multiparametric MRI (mpMRI) has better predictive value for the detection of clinically significant prostate cancer compared to traditional prostate-specific antigen (PSA) screening and allows better pre-biopsy risk assessment [23]. The combination of Prostate Imaging Reporting and Data System (PI-RADS) scores and patient age in the risk calculators significantly increases discriminative power for developing high-grade malignancy that can possibly reduce the number of unnecessary invasive procedures [24]. Sub-categorisation of patients into refined risk groups based on presenting PSA concentration, biopsy grade, and clinical stage enables more precise categorisation of low-risk and high-risk groups for death from cancer at diagnosis [25]. Incorporating AI algorithms into the interpretation of MRI for guided targeted biopsies helps to improve the specificity of the diagnosis and increase the clinician's confidence in identifying clinically significant lesions at treatment initiation [26].

Combining high-dimensional imaging features with clinical parameters and AI-based detection tools supports the move to an approach of accurate baseline risk stratification that better identifies aggressive phenotypes of the disease than does a single qualitative assessment [13,24,26]. Advanced computational frameworks that integrate multiparametric imaging data with traditional prognostic factors allow for the identification of select patient subgroups at risk for recurrence and/or mortality as part of more informed communication with regard to decision-making strategies in the escalation or lessening of therapy [19,25]. Table 2 summarises selected AI-supported detection, triage, and classification studies across mammography, MRI, ultrasound, and histopathology-related datasets, with reported performance varying by modality, dataset, task, and validation design.

**Table 2:** Baseline detection and diagnostic stratification CNN: convolutional neural network; DCNN: deep convolutional neural network; AUC: area under the curve; MRI: magnetic resonance imaging; mpMRI: multiparametric magnetic resonance imaging; RCT: randomised controlled trial; U-Net: U-shaped convolutional network; csPCa: clinically significant prostate cancer; bpMRI: biparametric magnetic resonance imaging; MVP: Multicenter Validation of Prostate Imaging; PI-RADS: Prostate Imaging Reporting and Data System; PPV: positive predictive value; DL: deep learning; GAN: generative adversarial network; DenseNet: densely connected convolutional network

Imaging modality	Dataset used	Model type	Task	Performance metrics	Key findings/predictors identified	Reference
Mammography	US and UK screening datasets	DeepMind DCNN	Detection	AUC improvement reported	Reduced false-positive and false-negative findings in breast cancer screening	[1]
Mammography	Danish Capital Region	Transpara DCNN	Detection/triage	AUC: 0.97	Reduced radiologist workload and false-positive findings	[6]
MRI (bpMRI)	MVP Study RCT	PI-RADS v2.1	Detection	PPV: 62.5% vs. 28.6%	Reduced biopsy rates and improved detection of csPCa	[23]
mpMRI	Peking University RCT	3D U-Net/AI-guided biopsy approach	Detection/biopsy guidance	csPCa detection rate: 58.64%	Improved detection of csPCa	[26]
Mammography/MRI/ultrasound/histopathology	Systematic review datasets	CNN, ensemble DL, GAN/DenseNet	Classification	Accuracy/AUC varied by modality	Summarised AI-based lesion classification and subtype prediction across imaging modalities	[4,27]

Early imaging indicators of therapeutic response

PET provides functional information on tumour metabolic activity and may detect treatment-related biological changes before clear anatomical changes are visible on CT or MRI [1]. Delta-radiomics measures changes in quantitative imaging features over time and may help identify early treatment response before size-based changes are captured by the conventional Response Evaluation Criteria in Solid Tumors (RECIST) assessment [14]. Deep learning-based radiomic biomarkers from thoracic CT scans have shown added prognostic value for immune checkpoint inhibitor response in advanced non-small cell lung cancer, including information beyond PD-L1 expression alone [11]. In immunotherapy, pseudoprogression can produce early inflammatory tumour enlargement, so functional and longitudinal imaging biomarkers may help distinguish true progression from immune-related treatment response [20].

AI-based response prediction has also been studied in colorectal and rectal cancer settings. A systematic review and meta-analysis reported that AI models may help predict response to chemotherapy or targeted therapy in metastatic colorectal cancer, although included studies varied in design, datasets, and validation methods [27]. AI-based quantification of tumour-infiltrating lymphocytes and mitotic index from histopathology slides has been evaluated for predicting chemoradiotherapy outcomes in rectal cancer [28]. MRI-based AI and radiomics models using post-treatment restaging scans have also been externally validated for predicting rectal cancer treatment response [29]. In colorectal cancer liver metastases, a multicentre cohort study showed that PET/CT-derived radiomic and clinical features could support prediction of bevacizumab treatment response and outcome [30].

Overall, multimodal imaging and pathology-based AI models may support earlier response assessment than size-based imaging alone. However, these findings should be interpreted as decision-support evidence, because model performance, validation settings, and clinical endpoints differ across tumour types and treatment contexts [11,14,27-30].

Longitudinal modelling of tumour evolution

Quantitative radiomic signatures can be tracked longitudinally during systemic therapy because interval changes may provide prognostic information that is not available from a single baseline scan [11,14]. Delta-radiomics measures changes in imaging features between time points and may help assess tumour heterogeneity, treatment response, and survival outcomes beyond static size-based measurements [14]. Dynamic risk recalibration in oncology should also consider temporal changes in tumour biology, molecular profile, and tumour microenvironment, particularly when treatment resistance or disease progression emerges during therapy [20]. Neural survival models that incorporate time-dependent information may help model non-linear relationships between treatment, patient characteristics, and outcomes across the clinical trajectory [17].

Multitask learning frameworks that analyse pretreatment and mid-treatment radiomic features together can capture temporal dependencies more effectively than models that treat each imaging time point as a separate task [31]. Radiomic features from multiple contrast phases, such as arterial and portal venous imaging, may support the assessment of early tumour evolution and recurrence risk in hepatocellular carcinoma [32]. Studies evaluating immunotherapy with transarterial chemoembolisation provide clinical context for changing tumour response patterns, although they are not direct radiomics model studies [33]. Siamese network architectures can compare paired longitudinal MR images and identify subtle imaging changes associated with therapeutic response after drug-eluting beads transarterial chemoembolisation (DEB-TACE) [34].

Overall, longitudinal modelling methods such as delta-radiomics, multitask learning, and Siamese networks may provide more clinically useful response information than single-time-point imaging alone, but their performance depends on tumour type, treatment setting, imaging protocol, and validation design [14,31,34]. Moving from baseline assessment to longitudinal risk monitoring requires imaging biomarkers to be analysed together with clinical and treatment variables over time, so that models can better reflect treatment resistance and disease progression during therapy [14,17].

AI in adaptive radiotherapy and surgical planning

Conventional radiotherapy planning is often affected by inter-observer variation during manual delineation of tumour volumes and organs at risk [1,35]. In retrospective imaging cohorts, radiomics-based models have shown potential for improving pre-treatment risk stratification, although this evidence is more directly related to prognosis than to contouring consistency [13]. Machine learning models trained on head and neck cancer datasets have been used to predict locoregional and distant recurrence patterns and may support treatment escalation or de-intensification decisions [36]. In MR-guided adaptive radiotherapy workflows, daily MRI acquired during treatment enables online plan adaptation by identifying anatomical changes between fractions and improving organ-at-risk sparing [36]. Dose-volume histogram analysis and related machine learning approaches have also been explored to identify radiation dose trade-off patterns and support personalised treatment planning [37-39].

Surgical planning is discussed only as a related example of AI-assisted anatomical and prognostic decision-making, not as the main focus of this section. In selected neuro-oncology contexts, multimodal neuroimaging features, including structural cortical thickness and functional connectivity, may support survival prediction and presurgical planning discussions [22]. Similarly, preoperative radiogenomic analysis of structural MRI may help predict molecular phenotype and inform surgical planning, although this evidence is distinct from adaptive radiotherapy planning [19]. Therefore, the main emphasis of this section remains on AI-supported radiotherapy planning, dose adaptation, organ-at-risk sparing, and longitudinal treatment monitoring.

Monitoring treatment-related toxicity and organ preservation

The qualitative interpretation of imaging by clinicians can be supplemented by AI tools that automate tumour and organ-at-risk delineation, helping assess how treatment may affect adjacent healthy tissues [1,36]. Multi-targeted AI algorithms applied to thoracic CT scans can simultaneously quantify cardiovascular, pulmonary, and skeletal imaging findings that may contribute to non-cancer mortality risk in patients with cancer [10]. In adaptive radiotherapy workflows, daily MRI enables the repeated assessment of organ-at-risk deformation and anatomical changes, including weight loss-related changes, to support treatment adaptation and reduce normal tissue exposure [36]. Dose-volume histogram analysis and automated dose-pattern assessment can help identify clinically relevant trade-offs between tumour target coverage and sparing of organs at risk, such as the parotid glands [37,40].

Machine learning classifiers using clinical, imaging, dosimetric, and radiomics features have been explored for the early prediction of chemoradiotherapy-induced cardiotoxicity in patients with breast cancer [38]. Multimodality cardiovascular imaging, including echocardiography and MRI, provides biomarkers such as global longitudinal strain and T2 relaxation time for detecting myocardial injury and inflammation during cancer therapy [40]. AI-based clinical decision aids linked with electronic health records may support shared decision-making by identifying patients at increased risk of cardiovascular complications after cancer treatment [41]. Machine learning models trained on clinical and echocardiographic data have also been evaluated for predicting cardiotoxicity and reduced cardiac function in breast cancer patients receiving anthracycline-based therapy [42].

**Figure 1:**
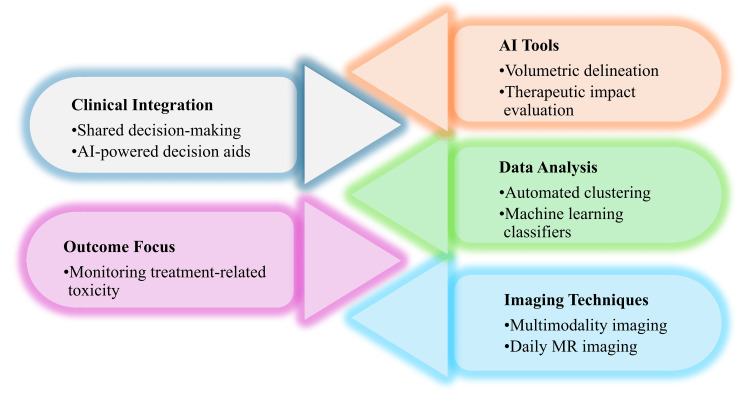
AI-driven framework for monitoring treatment-related toxicity during radiation oncology workflows The figure illustrates how AI supports treatment-related toxicity monitoring through the integration of imaging techniques, analytical methods, and clinical decision-support systems. Multimodality imaging and daily MRI provide longitudinal anatomical and functional data during treatment. AI tools, including volumetric delineation and therapeutic impact evaluation, assist in tumour and organ-at-risk assessment. Data analysis methods such as automated clustering and machine learning classifiers support the prediction of toxicity patterns and treatment response. These outputs are integrated into clinical decision-making workflows to support adaptive treatment planning and organ preservation. AI: artificial intelligence; MRI: magnetic resonance imaging Created by the authors using Microsoft PowerPoint (Microsoft Corporation, Redmond, Washington, United States)

Early identification of treatment-related toxicity may be improved by combining radiomic, dosimetric, cardiovascular imaging, and clinical variables within machine learning frameworks [38,40,42]. Predictive modelling of normal tissue complication probability, when combined with dose-volume assessment and AI-supported clinical decision tools, may help guide adaptive treatment modification and organ preservation during oncology care [10,36,37,41]. Figure 1 shows the AI-based framework for monitoring toxicity related to treatment using a combination of imaging techniques, analytical models, clinical decision systems, and prediction tools.

Real-time clinical implementation and decision support

Successful organisational readiness for AI deployment requires the alignment of high-bandwidth digital infrastructure with clinical leadership support and cross-functional change management [5]. Automating the initial interpretation of a medical image via computational tools frees up expert clinicians to reallocate their cognitive workload towards high-level management and decision processes during interventional management [8]. Shared decision-making supports through AI-powered decision aids that are integrated with health records identify high-risk patient trajectories for decision-makers in real-time [41]. The broad inclusion of prognostic biomarkers in clinical practice is currently limited by the interinstitutional lack of standardisation and variability of interpretation using different scanner technologies [1].

Clinician-friendly machine learning systems plugged into the standard Digital Imaging and Communications in Medicine (DICOM) formats and Picture Archiving and Communication System (PACS) viewers offer the support of real-time lesion detection in an existing radiological workflow [43]. Real-time intraoperative computer visualisation systems based on deep learning architectures significantly improve the decision-making time of tissue classification for frozen section analysis when compared to manual methods [44]. Multilayered AI frameworks provide disparate data fed from radiology platforms, genomic data and pathologic data, into decision-support systems to suggest transgene or classification outcomes with multidisciplinary tumour board recommendations [45]. The combination of deep learning tools with structured electronic health record data offers the potential for a powerful computational foundation for the personalisation of treatment strategies in prospective clinical trials [46].

**Figure 2:**
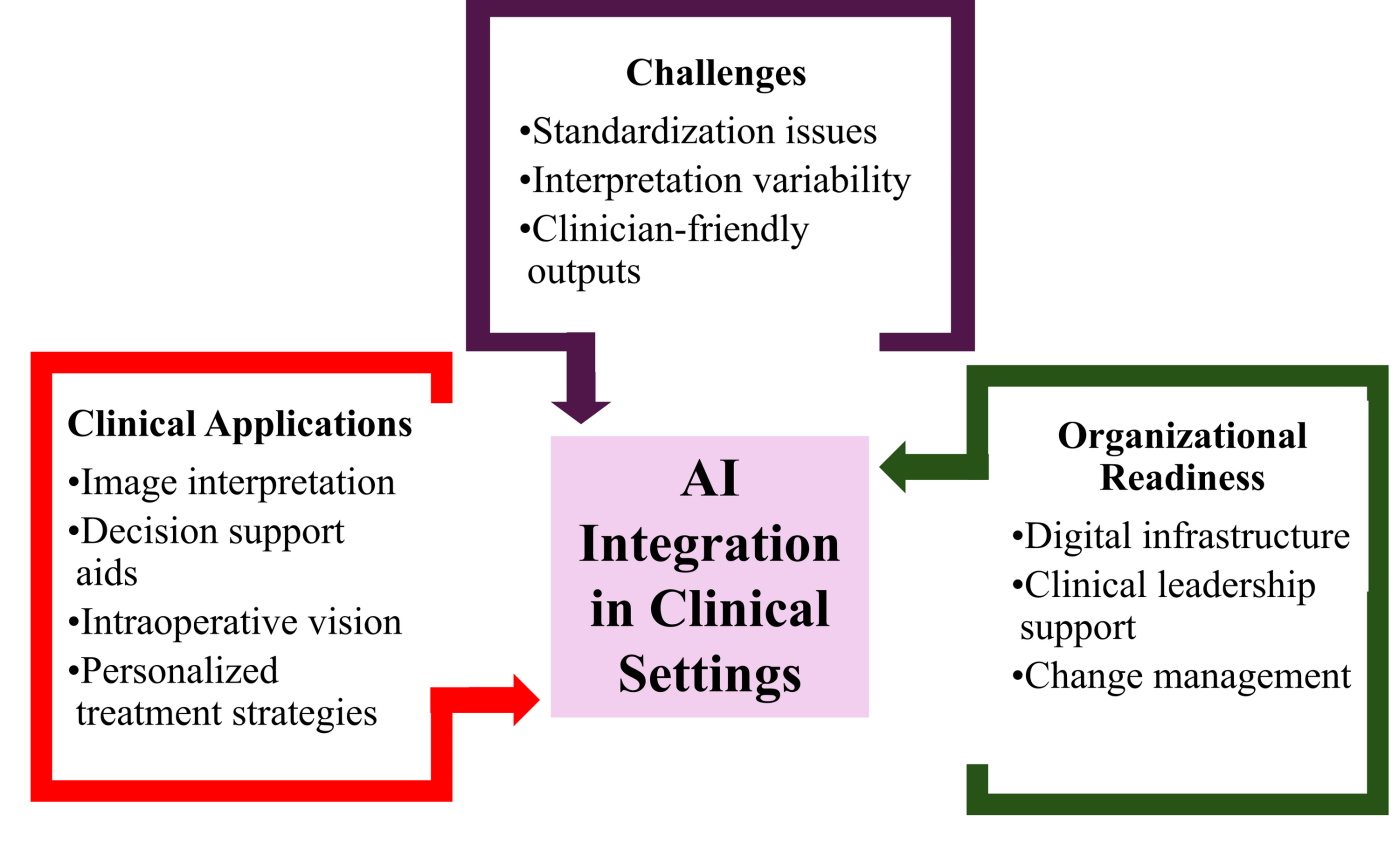
AI integration in clinical settings: challenges, readiness, and applications This figure shows how successful AI integration in clinical practice depends on the interaction between three domains. Clinical applications, including image interpretation, decision-support aids, intraoperative vision, and personalised treatment strategies, represent the intended uses of AI. Challenges such as standardisation issues, interpretation variability, and the need for clinician-friendly outputs may limit adoption. Organisational readiness, including digital infrastructure, clinical leadership support, and change management, is required to address these challenges and translate AI tools into routine clinical workflows. AI: artificial intelligence Created by the authors using Microsoft PowerPoint (Microsoft Corporation, Redmond, Washington, United States)

The transition of AI success from experimental data validation to epicentre clinical practice partitions demands the integration of clinician-friendly outcomes with standard clinical diagnostic platforms, minimising cognitive burdens and shortening the operative or diagnostic window of time [43,44]. The integration of multimodal prognostic signatures in multidisciplinary processes such as tumour board and shared decision-making platforms offers a permanent framework to recalibrate the clinical risk and select personalised therapies [45,46]. Figure 2 summarises the conceptual framework of the integration of AI in clinical settings, with a focus on major challenges, organisational readiness factors, and key clinical applications.

Comparative evaluation of AI-driven adaptive strategies versus conventional monitoring

Conventional radiotherapy planning is often affected by inter-observer variation during manual delineation of tumour volumes and organs at risk [1,36]. In retrospective imaging cohorts, radiomics-based models have shown potential for improving pre-treatment risk stratification, although this evidence is more directly related to prognosis than to contouring consistency [13]. Machine learning models trained on head and neck cancer datasets have been used to predict locoregional and distant recurrence patterns and may support treatment escalation or de-intensification decisions [35]. In MR-guided adaptive radiotherapy workflows, daily MRI acquired during treatment enables online plan adaptation by identifying anatomical changes between fractions and improving organ-at-risk sparing [36]. Dose-volume histogram analysis and related machine learning approaches have also been explored to identify radiation dose trade-off patterns and support personalised treatment planning [37,39].

Surgical planning is discussed only as a related example of AI-assisted anatomical and prognostic decision-making, not as the main focus of this section. In selected neuro-oncology contexts, multimodal neuroimaging features, including structural cortical thickness and functional connectivity, may support survival prediction and presurgical planning discussions [22,47]. Similarly, preoperative radiogenomic analysis of structural MRI may help predict molecular phenotype and inform surgical planning, although this evidence is distinct from adaptive radiotherapy planning [19,48]. Therefore, the main emphasis of this section remains on AI-supported radiotherapy planning, dose adaptation, organ-at-risk sparing, and longitudinal treatment monitoring [49,50]. Table 3 summarises AI-supported approaches for adaptive treatment monitoring, response prediction, toxicity assessment, and radiotherapy workflow optimisation. It distinguishes conventional imaging evidence from AI model performance to avoid presenting non-AI outcomes as algorithmic results.

**Table 3:** Adaptive treatment monitoring and toxicity prediction CT: computed tomography; AUC: area under the curve; PET: positron emission tomography; SPECT: single photon emission computed tomography; FLARE-RT: functional lung avoidance and response-adaptive escalation radiation therapy; SUVmax: maximum standardised uptake value; GLUT1: glucose transporter type 1; MRI: magnetic resonance imaging; AI: artificial intelligence; NLST: National Lung Screening Trial; DEB-TACE: drug-eluting beads transarterial chemoembolisation

Imaging modality/workflow	Dataset used	Model type	Task category	Performance metrics	Key findings/predictors identified	Reference
Low-dose CT	NLST	Conventional screening evidence, not AI model	Screening outcome context	20% lung cancer mortality reduction	Demonstrated the clinical value of structured imaging surveillance; not an AI performance metric	[1]
Thoracic CT	ARILIS cohort	Multi-target AI	Detection/risk quantification	AUC: 0.96-1.00	Cardiovascular and pulmonary quantification	[10]
CT	Head and neck cancer dataset	Machine learning algorithms	Clinical outcome prediction	Not consistently specified	Predicted radiotherapy outcomes in head and neck squamous cell carcinoma	[35]
MR-guided radiotherapy	Head and neck cohort	Adaptive radiotherapy workflow	Organ-at-risk sparing/adaptive planning	Not reported as AI model metric	Daily MR-guided adaptation supported organ-at-risk sparing	[36]
Head and neck radiomics	Systematic review	Radiomics/AI implementation review	Implementation barriers	Not applicable	Summarised challenges in AI-based radiomics implementation	[37]
Echocardiography/clinical data	Breast cancer cohort	Machine learning	Cardiotoxicity prediction	Model-dependent performance	Predicted cardiotoxicity in breast cancer patients receiving anthracycline	[42]
Clinical decision-support workflow	Cardio-oncology feasibility trial design	AI/patient similarity algorithms	Cardiovascular toxicity risk support	Feasibility outcome focus	Proposed AI decision aid for shared decision-making in cardiovascular toxicity prevention	[41]
PET/CT	Multicentre colorectal cancer liver metastases cohort	Deep radiomics-based fusion model	Bevacizumab response prediction	Study-reported response prediction metrics	Predicted treatment response and outcome using PET/CT-derived and clinical features	[30]
CT/PET/SPECT	FLARE-RT cohort	Multitask learning radiomics	Time-to-event prognostic modelling	PET C-index: 0.71	Multitask learning outperformed single-task modelling for survival prediction	[31]
MRI	Heidelberg cohort	Siamese network	Longitudinal response classification	AUC: 0.925	Dynamic tumour changes supported response prediction after DEB-TACE	[34]

Limitations and future recommendations

Although several multicentre cohorts, systematic reviews, and meta-analyses were included, the available evidence remains heterogeneous in methodology, validation design, and reporting quality across tumour types and imaging platforms. Current evidence is limited by frequent reliance on retrospective datasets, which reduces generalisability across different patient populations, scanners, treatment platforms, and institutional workflows. Many studies did not report a formal risk-of-bias assessment, and adherence to reporting standards for AI and radiomics studies was variable. Future reviews and primary studies should consider structured tools such as CLAIM, TRIPOD-AI, PROBAST, and radiomics quality assessment frameworks to improve transparency and reproducibility.

External validation was reported in some studies, but many models were tested only on internal cohorts or single-institution datasets, increasing the risk of overfitting. Prospective validation was less common, limiting confidence in real-world clinical performance. Algorithm performance may also vary because of differences in imaging acquisition, segmentation methods, contouring standards, radiomic feature extraction, and treatment planning systems. Limited interpretability of deep learning models may reduce clinician trust and complicate regulatory approval. Data privacy restrictions also limit large multi-institutional model development and benchmarking.

Future studies should prioritise prospective, multicentre validation across diverse demographic, technical, and clinical settings. Imaging acquisition, segmentation, contouring, and radiomic feature extraction pipelines should be standardised to improve reproducibility. Interpretable AI methods, federated learning, and systematic integration of genomic, clinical, dosimetric, and longitudinal imaging biomarkers should be evaluated to support safe clinical translation.

## Conclusions

This review synthesises current evidence on AI-driven prognostic modelling and adaptive treatment monitoring in radiation oncology, highlighting methodological advances and translational constraints. Integration of radiomics, machine learning, and multimodal clinical data demonstrates improved predictive stratification and dynamic response assessment compared with conventional approaches. Automated segmentation, longitudinal tumour tracking, and toxicity forecasting strengthen adaptive radiotherapy workflows and support individualised dose modulation. These developments expand the analytical capacity of routine imaging while enhancing clinical decision support. Sustained clinical integration depends on rigorous external validation, standardised imaging and feature extraction pipelines, and reproducible model development frameworks. This need is supported by the evidence summarised in the tables, where several models reported promising C-index, AUC, or accuracy values, but many were based on retrospective cohorts, single-institution datasets, or limited validation settings. Therefore, clinical adoption should depend not only on model performance but also on reproducibility, external validation, workflow compatibility, and demonstrated benefit in prospective radiation oncology settings. Limited multicentre prospective data and restricted interpretability remain barriers to regulatory approval and clinician confidence. Interoperability with existing treatment planning systems and secure data governance structures is required to ensure scalable deployment. Continued interdisciplinary collaboration among oncologists, physicists, and data scientists will be central to refining robust, transparent, and equitable AI systems capable of augmenting precision oncology while preserving clinical oversight and accountability.
